# Evolutionary clade affects resistance of *Clostridium difficile* spores to Cold Atmospheric Plasma

**DOI:** 10.1038/srep41814

**Published:** 2017-02-03

**Authors:** Mairéad Connor, Padrig B. Flynn, Derek J. Fairley, Nikki Marks, Panagiotis Manesiotis, William G. Graham, Brendan F. Gilmore, John W. McGrath

**Affiliations:** 1 School of Biological Sciences and the Institute for Global Food Security, Medical Biology Centre, Queens University Belfast, Northern Ireland; 2 School of Pharmacy, Medical Biology Centre, Queens University Belfast, Northern Ireland; 3 School of Mathematics and Physics, Queens University Belfast, Northern Ireland; 4 Department of Microbiology, Belfast Health & Social Care Trust, Belfast, Northern Ireland; 5 School of Chemistry and Chemical Engineering, Queens University Belfast, Northern Ireland

## Abstract

*Clostridium difficile* is a spore forming bacterium and the leading cause of colitis and antibiotic associated diarrhoea in the developed world. Spores produced by *C. difficile* are robust and can remain viable for months, leading to prolonged healthcare-associated outbreaks with high mortality. Exposure of *C. difficile* spores to a novel, non-thermal atmospheric pressure gas plasma was assessed. Factors affecting sporicidal efficacy, including percentage of oxygen in the helium carrier gas admixture, and the effect on spores from different strains representing the five evolutionary *C. difficile* clades was investigated. Strains from different clades displayed varying resistance to cold plasma. Strain R20291, representing the globally epidemic ribotype 027 type, was the most resistant. However all tested strains displayed a ~3 log reduction in viable spore counts after plasma treatment for 5 minutes. Inactivation of a ribotype 078 strain, the most prevalent clinical type seen in Northern Ireland, was further assessed with respect to surface decontamination, pH, and hydrogen peroxide concentration. Environmental factors affected plasma activity, with dry spores without the presence of organic matter being most susceptible. This study demonstrates that cold atmospheric plasma can effectively inactivate *C. difficile* spores, and highlights factors that can affect sporicidal activity.

*Clostridium difficile* is a Gram positive, spore forming, anaerobic bacterium and the leading cause of colitis and antibiotic associated diarrhoea in the developed world^1^. Spores produced by *C. difficile* are extremely robust and can remain viable for months, even in very harsh conditions such as high temperatures^2^ and biocidal challenge^3^. Their environmental stability and resistance allow them to persist until conditions become favourable for germination, for example, in the human gut. *C. difficile* spores have been found to remain on curtains, textiles, protective clothing and surfaces within hospital wards^4^. Transmission from fomites within healthcare settings has led to *C. difficile* overtaking methicillin resistant *Staphylococcus aureus* (MRSA) as the leading cause of hospital acquired infection globally^5^.

Symptoms of *C. difficile* infection (CDI) can range from mild to severe diarrhoea to pseudomembranous colitis, toxic megacolon, colonic perforation and death^6^,^7^. Certain factors increase the risk of CDI such as use of broad-spectrum antibiotics^8^, old age and hospitalization^9^. When an individual is diagnosed with a toxigenic *C. difficile* infection they are isolated, when possible, from other patients and facilities in an attempt to curb transmission via spores to other individuals in the hospital, particularly those on antibiotics^10^.

More than three hundred different ribotypes of *C. difficile* have been detected in humans^11^, with prevalence of different ribotypes varying between countries, and regionally^12^. Strains of *C. difficile* can be characterised into five genetically distinct clades, all of which contain toxigenic strains capable of causing infection^13^,^14^. In Northern Ireland the most common ribotype found to be causing infection in humans is ribotype 078^15^.

For decontamination of clinical areas, various methods are applied, including chemical disinfection, sporicidal fumigants and antimicrobial surfaces^16^. Many studies have shown that commonly used methods for hospital cleaning are not effective for the decontamination of *C. difficile* spores^17^, including alcohol based hand sanitation^18^. Chlorine based cleaning reagents, with a minimum of 1000ppm available chlorine, are capable of decontaminating fomites harbouring *C. difficile* spores^19^. However, UK COSHH regulations restrict occupational exposure to chlorine to 0.5ppm^20^. Difficil-S^®^ (Clinimax Ltd, UK) is a chlorine dioxide based sporicidal product which has found application in a number of healthcare settings, *C. difficile* eradication efficiency is nevertheless similar to that of standard National Health Service approved cleaning procedures^21^.

Sporicidal fumigation using gaseous hydrogen peroxide and chlorine dioxide may also be an effective strategy for *C. difficile* control^22^. Gaseous decontamination however requires rooms to be sealed for effective treatment, and the pre-cleansing of all surfaces. The effectiveness and commercial viability of such gas based decontamination strategies have yet to be fully established^22^. Furthermore when using gaseous chlorine dioxide, additional precautions must be taken, since it is explosive at high concentrations. Copper surfaces have also been found to reduce the viability of, but not fully eradicate, *C. difficile* spores^23^. Due to expense and efficacy, copper based systems can only be considered as part of a wider decontamination scheme^24^. There is therefore a clear need for robust alterative decontamination strategies for application within a clinical setting.

Atmospheric pressure non-thermal plasmas are attracting attention within the medical and life sciences fields as potential treatments for skin conditions, cancer, chronic infected wounds and decontamination of biotic and abiotic surfaces^25^. Atmospheric plasmas can be described as a partially ionised gas, which can produce a low-temperature plasma capable of propagating in air. This allows treatment and exposure of heat sensitive items at ambient pressure, without the necessity for large, bulky equipment, which is typically required for low-pressure systems. These low-pressure plasma systems have been employed within the medical setting for sterilization applications since the early 1970’s, although they could be more appropriately termed ‘plasma assisted’, combining the production of a plasma with addition of gaseous hydrogen peroxide^26^. In contrast plasma generated at atmospheric pressure by gases such as argon and helium can provide rapid on-site decontamination and could potentially be used for high-level disinfection in clinical environments.

This research employed an in-house designed kHz-driven dielectric barrier discharge plasma jet (Fig. 1) that has previously been shown to successfully exert rapid and significant antimicrobial activity against microorganisms in both planktonic and biofilm phenotypes^27^,^28^. In this study the sporicidal activity of the in-house system was evaluated, for the first time. Non-thermal plasma exposure against *C. difficile* spores was assessed and factors affecting sporicidal efficacy, including percentage of oxygen in the helium carrier gas admixture, and the effect of plasma treatment on spores from varying *C. difficile* strains (representing the five evolutionary *C. difficile* clades) was investigated. Inactivation of ribotype 078, the most prevalent clinical isolate in Northern Ireland, was further assessed with respect to surface decontamination, pH, and hydrogen peroxide concentration.

## Results

### Optimum oxygen concentration for deactivation of *C. difficile* spores with He/O_2_ plasma jet

In order to ascertain how the addition of oxygen affected plasma sporicidal activity, an oxygen titration was carried out to confirm the concentration of oxygen that would provide optimum spore inactivation. Between 0–0.5% oxygen gas added increased plasma-induced sporicidal activity (*p* = 0.005), with no further decrease noted after 0.5% (Fig. 2).

### Susceptibility of different *C. difficile* clades to non-thermal plasma exposure

Representative strains from the five evolutionary clades of *C. difficile* were exposed to cold plasma in quintuplicate: TL178 (clade 1, ribotype 002); R20291 (clade 2, epidemic ribotype 027); CD305 (clade 3, ribotype 023); CF5 (clade 4, ribotype 017) and M120 (clade 5, ribotype 078). Clade 1 and 5 isolates appear to be the most susceptible (Fig. 3A). After plasma exposure for 30 seconds the viable spores recovered for M120, TL178 and CD305 were 7%, 13% and 18% respectively, compared with CF5 and R20291 which had 40% and 52% viable spores remaining. All clades displayed approximately a 3 log reduction after plasma treatment for 5 minutes. The D value for each representative strain was different, with ribotype 027 (R20291, clade 2) proving to be the most resistant to the treatment (Table 1).

To exclude the possibility that cell debris in the spore preparations might influence susceptibility to plasma, spores from the most and least susceptible strains (M120 and R20291 respectively) were purified according to the method of Tavares *et al*.^29^ and subjected to plasma exposure (Fig. 3B). Cold plasma inactivation results using purified spore preparations were similar for both strains to those results obtained using the non-purified spore preparation (Fig. 3B). A D value of 0.4 minutes was seen for both purified and crude M120 spore suspensions, and with R20291 the D value for purified spores was 3.2 minutes compared to 3.4 minutes for non-purified spores.

### The effect of different environmental conditions on spore viability of strain M120

Deactivation of *C. difficile* ribotype 078 spores on surfaces was investigated under three different environmental conditions: spores dried onto the surface of microtitre plate wells; spores dried onto the surface in the presence of organic matter (0.03% BSA) and spores suspended in sterile distilled H_2_O. Spores dried onto the surface of the microtitre plate without organic matter appeared to be the most susceptible to plasma treatment with spore concentrations decreasing to 2.8 ± 1.2 spores per well after 1 min treatment (Fig. 4). With the spores in suspension, the numbers declined less rapidly but eventually reached an average of 25.4 ± 5.4 spores/100 μl after 5 minutes of plasma exposure, a 3 log reduction in spore count. For spores treated in the presence of BSA, spore counts declined to an average of 63.2 ± 16.6 spores per well after 5 minutes.

D values for plasma application in the different environments were calculated. Air-dried spores in wells were the most susceptible with a D value of 0.4 minutes. For both the spores in the presence of BSA and suspended in sterile H_2_O, the D values obtained were higher, being 1.7 and 1.8 minutes respectively.

### The effect of pH and H_2_O_2_ on spore viability of strain M120

Increasing plasma exposure time decreases water pH: After 2 and 5 minutes plasma exposure, pH decreases to pH 4 and pH 2 respectively (Fig. 5). After 2 minutes plasma exposure there was a significant drop in the number of spores recovered (*p* = 1.06 × 10^−9^). No further significant decrease in spore recovery was observed after 5 minutes treatment (*p* = 0.637; Fig. 5). In non-plasma treated, pH altered controls, exposure of spores to pH 4 for 2 minutes did not result in the same reduction in spore viability as with a 2 minute plasma exposure (*p* = 7.79 × 10^−7^); 12% of spores were recovered after plasma treatment compared to 41% in the pH4.0 amended cultures. Similarly in non-plasma treated pH 2.0 controls spore viability was significantly higher than in the corresponding plasma treated cultures (*p* = 2.37 × 10^−5^) (Fig. 6).

In addition to pH change, plasma treatment of water for 5 minutes results in the production of approximately 0.7 mM H_2_O_2_ (Fig. 5). Non-plasma treated pH 2 control cultures supplemented with H_2_O_2_ (0.7 mM) reduced spore viability less than the cognate 5 minute plasma treatment (*p* = 1.03 × 10^−9^; Fig. 6). There was no significant decrease in spore viability between pH 2 controls and those pH 2.0 controls augmented with H_2_O_2_ (0.7 mM). After exposure to plasma for 5 minutes, only 3% of spores were recovered. In non-plasma treated controls, after a 5 minute treatment at pH 2, or at pH 2.0 in the presence of H_2_O_2_ (0.7 mM), spore recoveries were 43% and 52% respectively (Fig. 6).

## Discussion

Previous studies have demonstrated the sporicidal effect of plasma produced in air with surface discharge^30^ and a compressed air jet^31^ to decontaminate *C. difficile* spores. The ability of a helium/oxygen atmospheric pressure plasma jet to deactivate *C. difficile* spores has also been demonstrated^32^, whilst a separate study found helium/oxygen plasma ineffective against *C. difficile* spores^33^. This study reinforces the fact that helium/oxygen plasma can be used for decontamination of *C. difficile* spores and describes for the first time how factors such as oxygen addition to the plasma, strain variation across the five genetic clades of *C. difficile* and environmental conditions can affect spore survival.

There was a noticeable increase in sporicidal activity with the increase in oxygen up to 0.5% with a plateau from the addition of 0.75% and 1% oxygen. The role of oxygen concentration in plasma-mediated sporicidal activity has been previously noted by Hong *et al*.^34^, whereby increasing oxygen concentration increased plasma activity against *Bacillus subtilis* endospores. That 0.5% oxygen added to plasma feed gas provided the greatest reduction would suggest the production of a particular species in the gas phase plasma may be responsible for plasma’s activity against *C. difficile* spores. Increasing oxygen past 0.5% may lead to a destabilisation of the plasma^35^.

The representative Clade 5/ribotype 078 strain M120 was the most susceptible to plasma exposure and recorded the shortest decimal reduction time for viable spore count (0.4 minutes). Ribotype 078 is the most common ribotype found infecting patients in Northern Ireland, and has been shown to be particularly virulent, with one study reporting 25% mortality within 14 days amongst infected individuals compared with 20% for 027 infections^36^.

Notably, the ribotype 027 isolate, R20291, showed an eight-fold increase in the D value and the greatest resistance to plasma treatment after 5 minutes exposure. The fact that *C. difficile* spores exhibit clade dependent tolerance to inactivation by plasma is an important observation. *C. difficile* ribotypes vary with respect to virulence, sporulation levels and toxin production rates^37^,^38^. Ribotype 027 appears to be particularly virulent, and has become a global epidemic strain^39^ causing numerous large outbreaks with high mortality. Conversely, there is evidence to suggest that not all strains of this ribotype can be categorised as highly virulent and that the clinical outcome is difficult to predict using ribotype alone^40^. Some studies have been carried out into the composition of the spore coat for *C. difficile*, in particular the role of the CotA protein, in spore resistance to environmental factors, such as heat and ethanol resistance^41^. Variation in spore coat structure may explain observed differences in susceptibility to cold plasma between strains.

The influence of different environmental conditions on plasma surface decontamination was investigated. *C. difficile* M120 spores were exposed to plasma under three different conditions: dried onto sterile plastic wells, dried onto sterile plastic wells in the presence of 0.03% BSA (replicating organic matter) and spores in aqueous suspension (replicating a wet/damp environment). A clean dry environment provided the greatest reduction in spores and shortest D value (0.4 minutes), compared to dried albumin (D value = 1.7 minutes) and *C. difficile* spores suspended in water (D value = 1.8 minutes). The observation that organic matter increases spore survival to plasma exposure has previously been reported by Klampf *et al*., 2014, where they assessed a surface micro-discharge against *C. difficile* endospores in the presence of 0.03% albumin^30^. The presence of organic matter is known to attenuate the efficacy of medical device disinfection^42^ and is an important consideration when assessing plasma for high level disinfection. In this scenario it is plausible that the BSA is providing a barrier between plasma exposure and the spores, or through sequestration of active species. That direct plasma treatment in a dry environment resulted in the greatest inactivation of spores suggests that surfaces and items for plasma disinfection would need to be clean and dry for effective decontamination. Further studies into factors that may affect spore inactivation would be justified, particularly with respect to spore age.

Comparing plasma exposures of spores in a dry and aqueous environment presents two differing plasma chemistries. In the dry environment effcacy would be highly dependent on the plasma’s gas phase reactive species and the electrostatic interaction of the plasma and the spores directly. In an aqueous environment the sporicidal activity would be dependent on either the generation of reactive species within the water, the reduction in pH of plasma treated water or a combination of both. With respect to the generation of reactive components, plasma treatment leads to the formation of both short lived (e.g. OH and ^1^O_2_), and long lived (hydrogen peroxide^26^) reactive species: Sun *et al*.^43^ have previously demonstrated that the former have half-lives in the range of 10^−6^–10^−9^ seconds (and would be both diluted and quenched upon plating of spores on solid media). Plasma-mediated hydrogen peroxide induced oxidative stress is therefore the primary hypothesised mechanism for the bactericidal nature of plasma^44^.

The effect of pH and hydrogen peroxide alone on sporicidal activity, in the absence of plasma treatment (Fig. 6), was investigated. Whilst pH appears to play a role in plasma-mediated spore inactivation, pH effects alone cannot fully explain the sporocidal activity of plasma. As inclusion of hydrogen peroxide into the pH 2 spore suspensions did not increase spore inactivation (in the absence of plasma), carry-over of hydrogen peroxide into the germination media - which would destroy *C. difficile* vegetative cells (i.e. through an indirect or delayed effect on viability) - seems unlikely. Further studies are thus required to dissect the mechanisms involved in plasma mediated sporicidal activity.

Low temperature plasmas are increasingly being utilized in clinical settings^24^,^45^,^46^ e.g. the portable kINPen system which was CE-certified in 2013. Our jet system has also previously been modified for hand-held use within the clinical environment. It is currently being assessed as a tool for the high level disinfection of niche areas, particularly the cantilever of duodenoscopes which are not amenable to traditional disinfectant methods^47^. This study therefore provides further evidence as to the potential of our cold plasma system for high level disinfection, in this case against *C. difficile*, a globally important spore-forming nosocomial pathogen.

## Materials and Methods

### Preparation of spore suspensions

Spore suspensions of representative ribotypes from the five *C. difficile* clades were prepared: - TL178 (Clade 1, Ribotype 002); R20291 (Clade 2, Ribotype 027); CD305 (Clade 3, Ribotype 023); CF5 (Clade 4, Ribotype 017) and M120 (Clade 5, Ribotype 078). All isolates were obtained from the Kelvin Laboratories at the Royal Victoria Hospital, Belfast. Brazier’s agar plates (OXOID, UK) were inoculated with each strain and incubated anaerobically (N_2_/CO_2_/H_2_) at 37 °C for 48 hours in an anaerobic chamber (Don Whitley Scientific). Colonies were harvested from the plates, resuspended in 5 ml sterile saline solution (Sigma-Aldrich Company Ltd., Dorset, UK) and subjected to alcohol shock (5 ml of absolute ethanol added to 5 ml suspension) for 1 hour to kill vegetative cells, in accordance with the spore culture method of UK Standards for Microbiology Investigations^48^. Suspensions were centrifuged at 3000 × g for 4 minutes, and washed once in 5 ml of sterile saline (0.9% w/v). Pellets were resuspended in 5 ml of saline (0.9% w/v), with Tween20 (0.05% v/v) (Sigma-Aldrich Company Ltd.).

#### Spore purification

Spore suspensions of R20291 and M120 were subject to further purification using the method of Tavares *et al*.^29^ giving spore preparations of >90% purity. Spore suspensions were centrifuged for 10 minutes at 10,000 × g at 4 °C and resuspended in 50 mM Tris-HCl (Sigma-Aldrich Company Ltd.), at pH 7.2, containing 50 μg/ml of lysozyme (Sigma-Aldrich Company Ltd.). After 1 hour incubation at 37 °C, the suspensions were centrifuged for 10 minutes at 10,000 × g and washed with 5 ml sterile distilled water. Suspensions were centrifuged again using aforementioned conditions and pellets were resuspended in 0.05% SDS solution. Spores were washed three times in sterile distilled water and final pellets were resuspended in 5 ml sterile distilled water. Spore purity was confirmed via phase microscopy.

### Preparation of test plate

#### For dry spores

Spore suspensions were centrifuged at 3000 × g for 4 minutes and spores resuspended in 70% ethanol. 100 μl aliquots of this ethanolic spore suspension were dispensed, with continuous mixing, into wells of a 96-well Nunc^TM^ microtitre plate (Sigma-Aldrich Company Ltd.) and left to air dry overnight in a fume cabinet (Holliday Fielding Hocking Ltd.).

#### For spores in suspension

Spore suspensions were centrifuged at 3000 × g for 4 minutes and resuspended in sterile dH_2_O. 100 μl aliquots of this aqueous spore suspension were dispensed, with continuous mixing, into wells of a 96-well Nunc^TM^ microtitre plate.

#### For spores in presence of organic matter

Spore suspensions were centrifuged at 3000 × g for 4 minutes and resuspended in an aqueous Bovine Serum Albumin, BSA, (Sigma Aldrich Corp.) solution at 0.03% (w/v)^30^. 100 μl aliquots of this BSA supplemented spore suspension were dispensed, with continuous mixing, into wells of a 96-well Nunc^TM^ microtitre plate and left to air dry overnight in a fume cabinet (Holliday Fielding Hocking Ltd.).

Each well contained 1300 ± 150 spores as determined by viable spore counts for each environmental condition. Each condition and exposure time was replicated five times.

### Plasma source and experimental plasma treatment of spores

**Figure 1:**
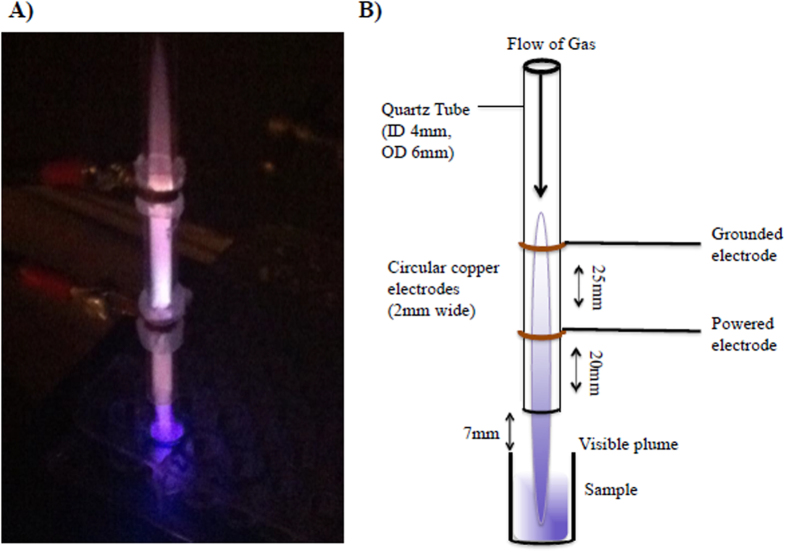
(A) Image of plasma treating a well of a 96 well plate. **(B)** Schematic diagram of plasma device.

**Figure 2:**
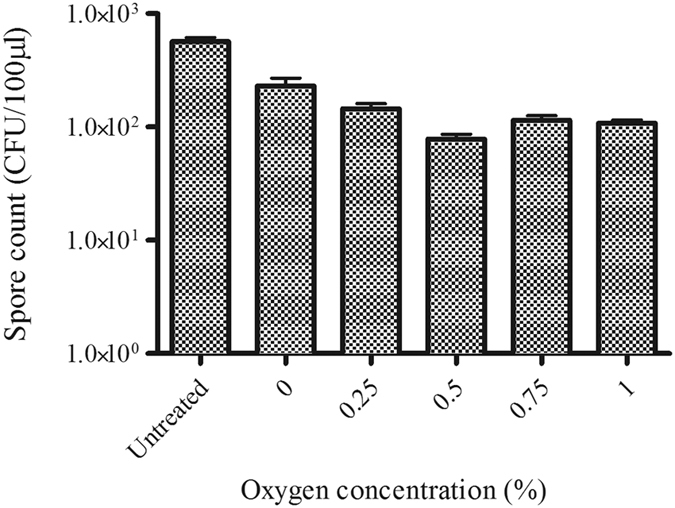
Survival of *C. difficile* spores after 60 seconds plasma exposure using varying oxygen concentrations within a helium plasma alongside untreated spores. Each bar represents the average of five replicates with error bars showing the standard error.

**Figure 3:**
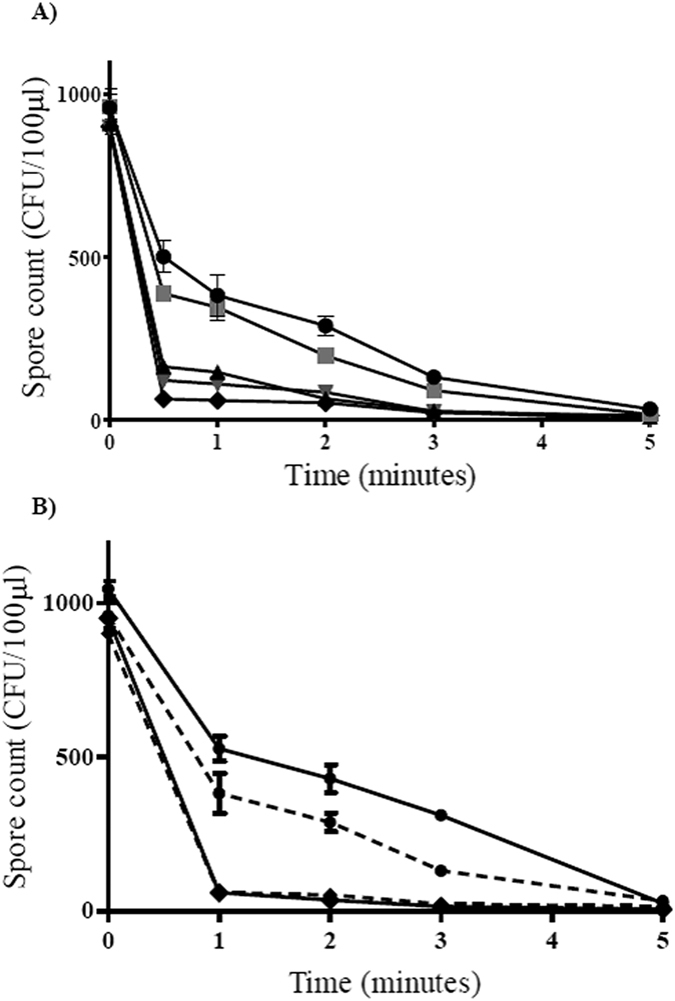
(**A**) Comparison of clade-specific tolerance to atmospheric pressure plasma treatment; R20291(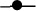), CF5 (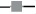), CD305 (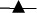), TL178 (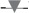) and M120 (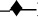). Spores were dried onto wells of a 96 well plate and treated with a helium/oxygen (0.5%) plasma plume. Each bar represent the average of five replicates, error bars represent the standard error; (**B**) Effect of He/O_2_ plasma on purified and non-purified spores from most and least resistant strains, R20291 and M120. The plasma jet was used to treat purified spores dried onto microtitre plate wells from R20291 (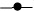) and M120 (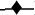) and non- purified spores dried onto microtitre plate wells from R20291 (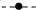) and M120 (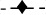). Each bar represents the average of three replicates, error bars represent the standard error.

**Figure 4:**
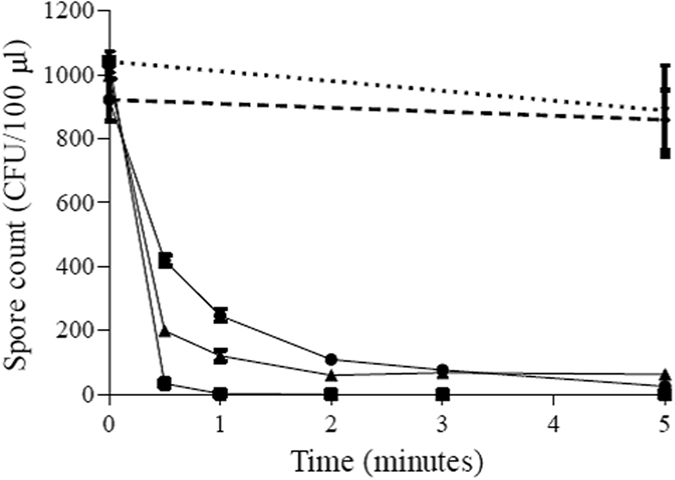
Effect of He/O_2_ plasma on *C. difficile* M120 spores under different conditions. The plasma jet was used to treat spores dried onto microtitre plate wells (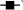), spores dried in the presence of 0.03% BSA (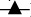), and spores suspended in sterile distilled H_2_O (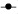). Gas-only controls, both on dry spores (small dash) and in suspension (large dash), are also displayed. Each point shows the average of five replicates along with the associated standard error.

The atmospheric pressure kHz plasma source used in this study has previously been described in detail^28^,^49^,^50^. Developed within the School of Mathematics and Physics Queen’s University Belfast, the jet consists of two copper electrodes encircling a quartz tube with an inner diameter of 4 mm and outer diameter of 6 mm. The powered electrode is 20 mm from the nozzle end and the grounded electrode is 25 mm from the powered. Standard operating conditions of 6 kV and 20 kHz were used to produce a helium/oxygen (0.5%) plasma with a gas flow rate of 2 standard litre per minute (SLM). Samples were treated within wells of a 96 well plate at a distance of 7 mm from the end of the quartz tube to the top of the plate as shown in the experimental schematic (Fig. 1). The top of the 96 well plate was at a minimum distance of 27 mm from the powered electrode.

For each treatment condition and treatment time, five replicates were exposed to the plume of the plasma jet for 0, 0.5, 1, 2, 3 and 5 minutes within the wells of a 96 well plate. Helium/oxygen (0.5%) gas at a flow rate of 2 SLM was used to treat dried spores for 5 minutes ensuring that gas flow alone did not affect spore counts within the wells (gas-only controls).

The time required to achieve a 1 log reduction in viable spore count (D, minutes) was calculated for each experimental condition.

After plasma exposure experiments, Brazier’s agar plates (OXOID, UK) were inoculated with the 100 μl spore suspensions (either taken directly from wells for aqueous spore suspensions, or after resuspension of dry spores using 100 μl of sterile distilled H_2_O) and incubated at 37 °C in an anaerobic chamber for 48 hours. After incubation viable spore counts were obtained by counting the colonies formed on the agar plate.

### Measurement of hydrogen peroxide and pH of plasma treated water

Unless otherwise specified, reagents and chemicals were supplied by Sigma-Aldrich. Titanium sulphate was used to colorimetrically determine the concentration of hydrogen peroxide produced in plasma treated water^51^,^52^. 100 μl of deionised water was exposed to the plasma effluent for the same time points as for the spore exposure in triplicate. After exposure 10 μl was added to 90 μl of water in wells of a Nunc-96 well plate (Fisher Scientific UK Ltd. Loughborough, UK) followed immediately by the addition of 10 μl of a 60 mM sodium azide solution to stabilize hydrogen peroxide in acidic conditions. 50 μl of titanium sulphate was added forming the yellow peroxotitanium complex. Absorbance was read at 400 nm using a microplate reader. (BioTek EL808; BioTek Instruments Ltd. Potton, UK). H_2_O_2_ concentrations were calculated from a standard curve. pH measurements of plasma treated water were conducted in triplicate using a calibrated Minitrode combination electrode pH meter (Hamilton Company, Bonaduz, Switzerland).

### Effect of pH and Hydrogen Peroxide on spore viability of M120

The effect of pH and hydrogen peroxide (H_2_O_2_) production on spore viability was investigated as they represent known conditions induced by plasma exposure^51^.

Solutions of sterile distilled water at pH 2, 4 and 7 were prepared, the pH was adjusted using 0.1 M hydrochloric acid (HCl) alongside a 700 μM solution of hydrogen peroxide at pH 2.

**Figure 5:**
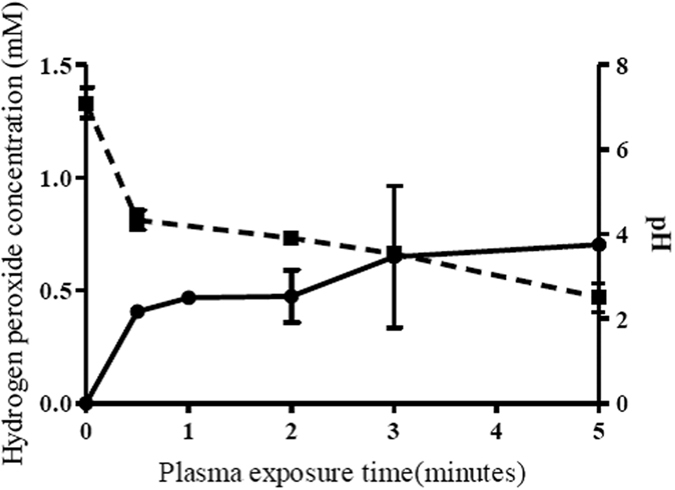
Effect of plasma exposure on 100 μl of water on pH (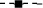) and the production of hydrogen peroxide (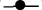). Each point represents the mean of three replicates with standard error.

**Figure 6:**
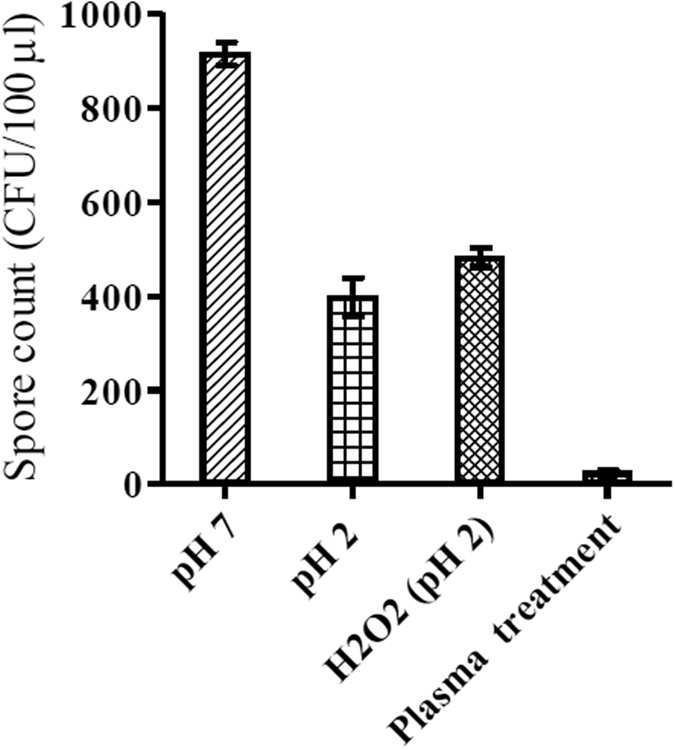
The effect of pH, hydrogen peroxide concenration and the corresponding plasma treatment of 5 minutes on viable spore count. Each bar represents the average of nine replicates with error bars showing the standard error.

Aliquots of *C. difficile* M120 spore suspension were centrifuged at 3000 × g for 4 minutes and spores resuspended in the appropriate test solutions. Spores were incubated at each pH for times equivalent to cold plasma treated water (Fig. 5) to control for sporicidal effects due to low pH and H_2_O_2_ production alone.

## Additional Information

**How to cite this article:** Connor, M. *et al*. Evolutionary clade affects resistance of *Clostridium difficile* spores to Cold Atmospheric Plasma. *Sci. Rep.* **7**, 41814; doi: 10.1038/srep41814 (2017).

**Publisher's note:** Springer Nature remains neutral with regard to jurisdictional claims in published maps and institutional affiliations.

## Figures and Tables

**Table 1:** Summary of D values for plasma treatment of each representative strain of the five clades.

Strain (Ribotype, Clade)	D value (minutes)
M120 (078, 5)	0.4
CD305 (023, 3)	1.9
TL178 (002, 1)	2.3
CF5 (017, 4)	3.2
R20291 (027, 2)	3.4
